# Structure and Organisation of SinR, the Master Regulator of Biofilm Formation in *Bacillus subtilis*

**DOI:** 10.1016/j.jmb.2011.06.004

**Published:** 2011-08-19

**Authors:** Vicki L. Colledge, Mark J. Fogg, Vladimir M. Levdikov, Andrew Leech, Eleanor J. Dodson, Anthony J. Wilkinson

**Affiliations:** 1 Structural Biology Laboratory, Department of Chemistry, University of York, York YO10 5YW, UK; 2 Molecular Interactions Laboratory, Department of Biology, University of York, York YO10 5YW, UK

**Keywords:** SEC-MALLS, size-exclusion chromatography with multi-angle laser light scattering, ESRF, European Synchrotron Radiation Facility, PDB, Protein Data Bank, HTH, helix–turn–helix, EDTA, ethylenediaminetetraacetic acid, SPR, surface plasmon resonance, PEG, polyethylene glycol, SeMet, selenomethionine, HEX, hexachlorofluorescein, biofilm regulation, *Bacillus subtilis*, SinR, crystal structure, repressor

## Abstract

*sinR* encodes a tetrameric repressor of genes required for biofilm formation in *Bacillus subtilis*. *sinI*, which is transcribed under Spo0A control, encodes a dimeric protein that binds to SinR to form a SinR–SinI heterodimer in which the DNA-binding functions of SinR are abrogated and repression of biofilm genes is relieved. The heterodimer-forming surface comprises residues conserved between SinR and SinI. Each forms a pair of α-helices that hook together to form an intermolecular four-helix bundle. Here, we are interested in the assembly of the SinR tetramer and its binding to DNA. Size-exclusion chromatography with multi-angle laser light scattering and crystallographic analysis reveal that a DNA-binding fragment of SinR (residues 1–69) is a monomer, while a SinI-binding fragment (residues 74–111) is a tetramer arranged as a dimer of dimers. The SinR(74–111) chain forms two α-helices with the organisation of the dimer similar to that observed in the SinR–SinI complex. The tetramer is formed through interactions of residues at the C-termini of the four chains. A model of the intact SinR tetramer in which the DNA binding domains surround the tetramerisation core was built. Fluorescence anisotropy and surface plasmon resonance experiments showed that SinR binds to an oligonucleotide duplex, 5′-TTT**GTTCTCT**AA**AGAGAAC**TTA-3′, containing a pair of SinR consensus sequences in inverted orientation with a *K*_d_ of 300 nM. The implications of these data for promoter binding and the curious quaternary structural transitions of SinR upon binding to (i) SinI and (ii) the SinR-like protein SlrR, which “repurposes” SinR as a repressor of autolysin and motility genes, are discussed.

## Introduction

Many bacteria are able to form architecturally complex communities of cells called biofilms.^1^ During biofilm formation, bacteria attach to a surface and secrete proteins and polysaccharides that create a protective and stabilising matrix surrounding the cells. As biofilms can form on almost any surface, they are a concern in human health and disease, notably when they form on implanted medical devices.^2^ The presence of the biofilm matrix confers a degree of resistance to antimicrobial agents, further complicating treatments. For *Bacillus subtilis*, which has been used as a model organism to study cellular differentiation during biofilm formation, biofilms can also take the form of floating structures called pellicles, which appear at air–liquid interfaces.^3^ In this case, a submerged motile population of single cells switches to a state in which the cells grow as chains that become bundled and rise to the surface.

The master regulator of biofilm formation in *B. subtilis* is the transcriptional repressor SinR,^4^ and SinI is its antagonist. The two proteins were earlier associated with *s*porulation *in*hibition and regulation of extracellular protease production^5,6^ in “domesticated” laboratory strains of *B. subtilis.* These strains have lost the ability to form robust biofilms, in contrast to wild strains that readily do so. In undomesticated strains, it was shown that deletion of *sinI* prevented biofilm formation, while *sinR* null mutants readily formed multicellular structures.^4^ SinR was shown to repress the transcription of the *epsA-O* operon encoding enzymes for expolysaccharide biosynthesis and the *yqxM-sipW-tasA* operon encoding TasA, which forms amyloid-like fibres that bind cells together in the biofilm.^4,7–9^

**Fig. 1:**
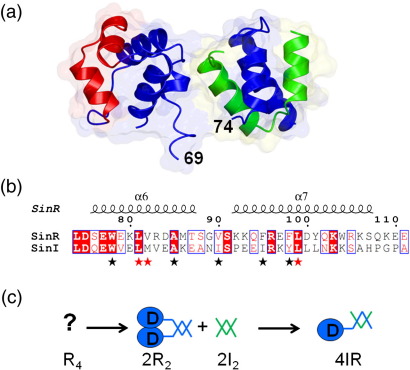
SinI–SinR interaction. (a) Structure of SinR in complex with SinI shown in ribbon representation. SinR is shown in blue, with the HTH (residues 17–36) in red. SinI is shown in green. Residues 70–73 of SinR are missing from the structure. The proteins form a heterodimer through interactions of the SinR C-terminal region, which acts as a two-helical hook interacting with the similarly structured SinI. The translucent surface emphasises the intimacy of the interaction. This and other structure images were generated in CCP4MG.^16^ (b) Sequence alignment of the two-helical hook regions of SinR and SinI. Identical residues are highlighted red, with similar residues boxed. Asterisks below the alignment emphasise the conservation of a series of apolar residues that are integral to (black) or surrounding (red) the intermolecular hydrophobic core. Secondary structure elements and residue numbering above the alignment refer to SinR. The alignment was created using ClustalW and ESPript.^17^ (c) Model of the quaternary structure changes accompanying formation of the SinI–SinR complex and models of SinR dimers and SinI dimers based on the sequence conservation and interactions observed in the structure of the SinR–SinI heterodimer. The DNA binding domain of SinR is represented as a blue circle and labelled D; the two-helical hooks of SinR (blue) and SinI (green) are represented as V-shapes.

The genes encoding SinR (111 residues) and SinI (57 residues) are adjacent on the *B. subtilis* chromosome.^10,11^ *sinR* is constitutively expressed, while *sinI* is expressed under the control of Spo0A, a response regulator that is activated by a multicomponent phosphorelay during stationary phase.^10,12^ Upon mixing of the purified proteins, there is a rearrangement of dimeric SinI and tetrameric SinR to form a SinI–SinR heterodimer.^13,14^ This striking quaternary reorganisation abolishes the capacity of SinR to bind to DNA and to inhibit the transcription of its target genes. The crystal structure of SinI–SinR revealed two domains connected by a short linker that was not visible in the electron density maps^15^ (Fig. 1a). The first domain is made up of residues 1–69 of SinR arranged in a helical bundle that contains a helix–turn–helix (HTH) motif (residues 17–36). This domain has close structural similarity to the DNA binding domains of the repressor proteins of λ-type bacteriophages. The second domain is made up of residues 74–111 of SinR and residues 3–39 of SinI, which form an intermolecular four-helix bundle in which each chain contributes a pair of helices that hook together (Fig. 1a). Two aspects of this structure are of note. The first is that the heterodimerisation domain has a striking hydrophobic core. The second is that the residues that contribute to this hydrophobic core are conserved in the sequences of SinI and the C-terminal region of SinR (Fig. 1b). This led us to speculate that the two-helical hook regions of the respective proteins might be used to form homodimer interfaces in SinI and SinR in analogous but evidently less stable intermolecular hydrophobic cores (Fig. 1c).

The renaissance of SinR as a repressor of biofilm formation prompted us to revisit the question of the organisation of the SinR tetramer and how it might bind to DNA. Analysis of the new found SinR target promoters *PepsA-O* and *PyqxM* led to the identification of a convincing 7-bp DNA binding consensus sequence, GTTCTYT.^7^ This sequence occurs in multiple copies and in a variety of orientations at these promoters, and it is also present upstream of *aprE*, the gene encoding the extracellular protease subtilisin.^7,18^ The interest in SinR is heightened by the recent discovery that it binds to SlrR, a SinR-like protein.^19^ Sequence comparisons suggest that SlrR, too, possesses a two-helical hook oligomerisation domain.

Here, we have characterised the affinity and stoichiometry of DNA binding by SinR using oligonucleotide ligands containing different arrangements of the consensus sequence using biophysical methods. We show using hydrodynamic methods that the C-terminal domain of SinR is responsible for dimer and tetramer formation and, using crystallographic methods, that pairs of SinR chains associate using their two-helical hooks to pack together to form a hydrophobic core. The extreme C-terminal regions of the four SinR chains in two dimers subsequently mediate tetramer formation. A model of an intact SinR tetramer is presented and discussed in terms of its implications for DNA and SlrR binding.

## Results

### SinI–SinR interaction examined by light scattering

**Fig. 2:**
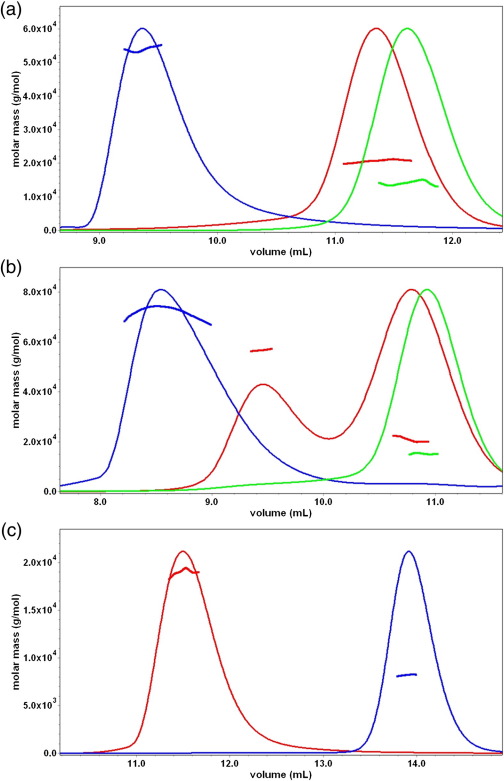
Molecular mass measured from SEC-MALLS analysis. In (a) to (c), the thinner lines trace the absorbance at 280 nm of the eluate from a Superdex 10/30 S75 column as a function of elution volume. The thicker lines represent the weight-average molecular weight of the species in the eluate calculated from refractive index and light-scattering measurements. (a) Overlay of chromatograms of SinR (blue), SinI (green) and an equimolar mixture of SinI and SinR (red). (b) Analysis of SinR binding to DNA. The chromatograms are associated with the  22-bp oligonucleotide containing a tandem repeat of the SinR binding motif (green), which gives a molecular mass value of 15 kDa. The red trace is a chromatogram of a sample of SinR mixed with this oligonucleotide. It exhibits two peaks corresponding to SinR (molecular mass = 57 kDa) and DNA (molecular mass = 21 kDa). The molecular mass of the DNA peak seen in this mixture is overestimated because of a tail of co-eluting protein that is not strongly represented in the UV trace. The blue trace is a chromatogram of SinR mixed with a  22-bp DNA duplex containing a pair of SinR binding motifs in inverted orientation. (c) Analysis of the domain fragments of SinR. A trace of histidine-tagged SinR(1–69) in blue is shown together with a trace derived from a sample of SinR(77–111).

The interactions of SinI and SinR were reexamined in a size-exclusion chromatography with multi-angle laser light-scattering (SEC-MALLS) experiment. In these experiments, samples are fractionated on a gel-filtration column, and the absorbance at 280 nm and the refractive index of the eluate are monitored together with the multi-angle laser light scattering of the sample. This enables the weight-average molecular weight (M_w_) of species in the eluate to be calculated continuously. SinR, SinI and an equimolar mixture of the two proteins were analysed, and the chromatograms are presented in Fig. 2a. For SinR and SinI, the *A*_280_ traces show single peaks associated with calculated molecular masses of 54 kDa and 14 kDa, respectively, consistent with the presence of SinR tetramers (expected molecular mass = 53 kDa) and SinI dimers (expected molecular mass = 14 kDa). Following mixing, a new species appears with a mass of 21 kDa, corresponding to the SinR–SinI heterodimer (expected  molecular mass = 20 kDa). For SinR and the SinI–SinR complex, the straightforward SEC-MALLS analysis reproduces the results of the earlier extended analysis using analytical ultracentrifugation.^13^

### SinR binds to oligonucleotides containing the consensus sequence

**Fig. 3:**
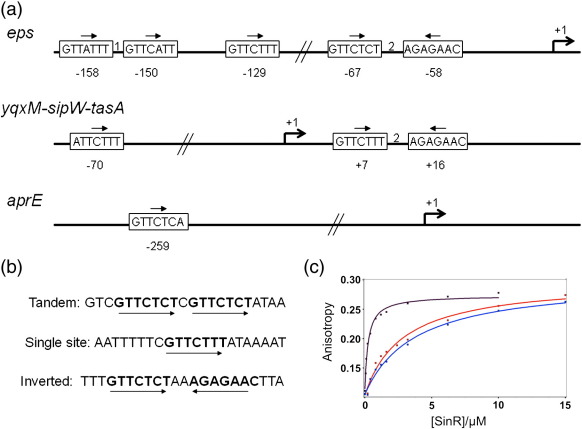

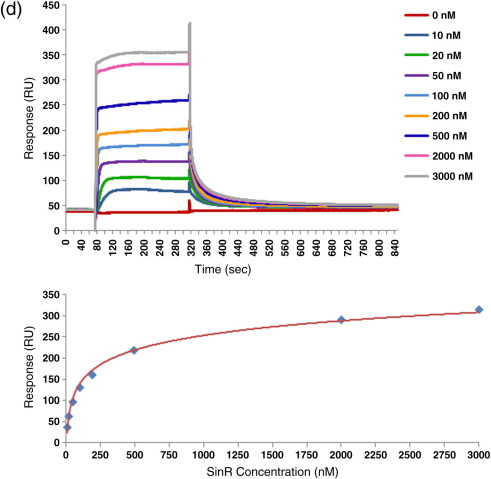
SinR binding to DNA. (a) The arrangement of SinR binding motifs at the *B. subtilis eps*, *yqxM* and *aprE* promoters. SinR binding sites are shown as boxes, with the putative SinR binding sequence displayed. The relative orientation of the sequences is shown by the arrows. Numbers between boxes indicate the number of base pairs between the sequences. Numbers below the boxes represent their position with respect to the transcription start site at + 1, which is indicated by an arrow. (b) Oligonucleotide sequences used for DNA binding studies in this work. The double-stranded oligonucleotide duplexes contain the 7-bp SinR binding motifs, highlighted in bold, in three arrangements/orientations. (c) Fluorescence anisotropy measurement of SinR binding to 22-bp oligonucleotide duplexes. The binding curves show an increase in the anisotropy of the fluorescence of the labelled DNA upon successive addition of SinR. The calculated anisotropy values (squares) were fitted to a model (line) using the Scientist software. The lines in purple represent the inverted repeat, the lines in blue represent the tandem repeat and the lines in red represent the single-site duplex. SinR binds with 10-fold higher affinity to the inverted repeat sequence and with similar lower affinity to the tandem repeat and single-site sequences. (d) SPR analysis of SinR binding to the inverted repeat DNA duplex. The curves show an increase in response units (RU) as SinR binds to DNA, followed by a decrease as the complex dissociates. The SinR injection began at 80 s and ended at 320 s. Each sensorgram is a binding experiment carried out at a different SinR concentration in the range 0–3000 nM. Data from the SPR sensorgrams were used to plot a steady-state binding curve for the interaction of SinR with the inverted repeat oligonucleotide. The point corresponding to 0 nM SinR was set to 0 RU, and all other data points were scaled to this. The point at 0 nM SinR and 0 RU is omitted.

Examination of the *eps*, *yqxM* and *aprE* promoters revealed a consensus sequence, 5′-GTTCTYT-3′, within the region of the DNA protected by SinR in DNase I footprinting experiments.^7,18^ The sequence motif occurs in different numbers and orientations at the different promoters (Fig. 3a).

To investigate the binding of SinR to DNA, we designed 22-bp oligonucleotide duplexes containing the SinR consensus binding motif (SinRBM). Two of these consisted of a pair of SinRBMs in inverted repeat and tandem repeat combinations, and the third contained a single SinRBM (Fig. 3b). We examined the binding of SinR (1–5 μM) to the three duplexes (0.5 μM) in gel electrophoretic mobility shift experiments. The mobility of all three duplexes was decreased in the presence of SinR (data not shown), with the inverted repeat duplex exhibiting higher affinity. In contrast, we saw no gel mobility shift with a  22-bp duplex that lacked SinRBMs. For each of the duplexes, one of the strands was 5′ labelled with hexachlorofluorescein (HEX) so that DNA binding could be monitored quantitatively by fluorescence anisotropy.^20^ Addition of SinR to each DNA ligand was accompanied by an increase in fluorescence anisotropy, indicating the formation of higher molecular species as the protein binds to its target. The anisotropy changes accompanying titration of the DNA with SinR were plotted as a function of the SinR concentration, and binding curves fitted to these data were used to estimate the dissociation constants. The binding curves, shown in Fig. 3c, show that SinR binds with the highest affinity (*K*_d_ = 300 nM) to the duplex containing the inverted repeat. The duplexes containing the tandem repeat and single-site sequences have similar *K*_d_ values, 3.7 μM and 2.8 μM, respectively, which are 10-fold higher than that for the inverted repeat duplex. For the inverted repeat duplex, analysis of a plot of anisotropy *versus* log[SinR] (not shown) revealed no evidence to suggest that the binding of SinR is cooperative.

Surface plasmon resonance (SPR) was used as an independent method of measuring the affinity of SinR for the inverted repeat DNA. For these experiments, one of the strands was biotinylated on its 5′ end so that the duplex could be immobilised on a streptavidin sensor chip. SinR was flowed over the chip at a range of concentrations, and the time courses of association and dissociation were monitored. Sensorgrams are overlaid in Fig. 3d, which also shows a plot of the amplitude of the change in response units as a function of the SinR concentration. This steady-state affinity method of analysis yielded a *K*_d_ value of 270 ± 50 nM, in good agreement with the results from fluorescence anisotropy. The association rates were very high, such that the data could not accurately be analysed with kinetic models, and thus, association and dissociation rate constants were not determined from these data.

Finally, we examined by SPR the effect of SinI on the interaction of SinR with DNA. Pre-incubation of SinR with increasing concentrations of SinI, prior to flowing the protein mixture over the DNA immobilised on the sensor chip surface, led to decreases and, above stoichiometric ratios, abolition of the rise in response units, indicating inhibition of SinR binding to DNA (data not shown). As previously inferred, the SinI–SinR complex is not competent to bind to DNA or inhibit transcription.^11,15^ Interestingly, binding of SinR to immobilised DNA followed by passage of SinI over the chip did not remove SinR from the DNA, suggesting that the formation of the SinR–SinI complex is relatively slow.

### SinR binds to DNA as a tetramer

To investigate the stoichiometry of the SinR–DNA complex, we performed a SEC-MALLS experiment. Runs were carried out on samples of SinR mixed with oligonucleotides containing the inverted and tandem repeats of the SinRBM as well as on samples of the free oligonucleotides and protein. SEC-MALLS analysis of the DNA fragments (illustrated for the tandem repeat oligonucleotide in Fig. 2b) showed a principal peak associated with a mass of 15 kDa corresponding to double-stranded DNA (calculated mass, 14 kDa). A mixture of the inverted SinRBM repeat oligonucleotide and SinR at a 1:1 ratio of SinR tetramer to DNA duplex produced a principal *A*_280_ peak with lower retention time than either of the component species, and this peak was associated with a higher molecular mass value of 74 kDa, as shown in Fig. 2b. This value is likely to represent a mixture of the SinR tetramer bound to one DNA molecule (expected mass of 67 kDa) and the SinR tetramer bound to two DNA molecules (expected mass of 81 kDa).

We repeated these experiments with SinR and the tandem repeat SinRBM DNA (Fig. 2b). In this experiment, the *A*_280_ peaks are associated with species with molecular mass values of 57 kDa and 21 kDa, indicating that the protein and DNA components were resolved on the column and that there was no evidence of complex formation (data not shown). This is presumably because this complex has a 10-fold higher *K*_d_ and there is extensive dissociation of the complex as it flows through the column.

### SinR(1–69) is a monomer in solution that dimerises in the crystal

To establish the determinants of quaternary structure in SinR, we expressed separately fragments of *sinR* encoding the DNA binding domain (residues 1–69) and the SinI binding domain (residues 74–111). SEC-MALLS analysis of SinR(1–69) gave a molecular mass value of 8 kDa (Fig. 2c), close to the calculated molecular mass of the tagged polypeptide chain (8.9 kDa) and indicating that the DNA binding domain fragment is a monomer, in agreement with earlier measurements.^13^

Crystals of SinR(1–69) were used for X-ray diffraction data collection at the European Synchrotron Radiation Facility (ESRF), Grenoble, and the structure was solved to 1.9-Å spacing by molecular replacement (Table 1). The two chains in the asymmetric unit, A and B, are closely superimposable [root-mean-squared displacement (rmsΔ) = 0.4 Å for 65 equivalent C^α^ atoms]. The structure of the DNA binding domain is essentially identical in crystals of SinR(1–69) and in those of the SinR–SinI complex. Equivalent C^α^ atoms in the two structures can be superposed with an rmsΔ of 0.5 Å, indicating that the binding of SinI does not lead to changes in the structure of the DNA binding domain of SinR.

**Fig. 4:**
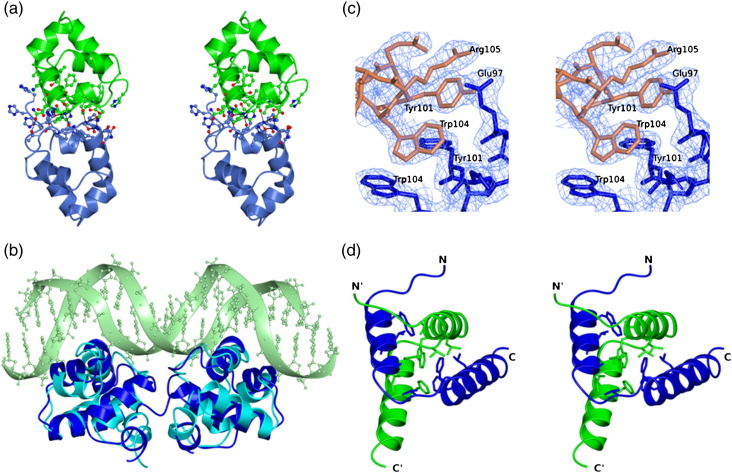

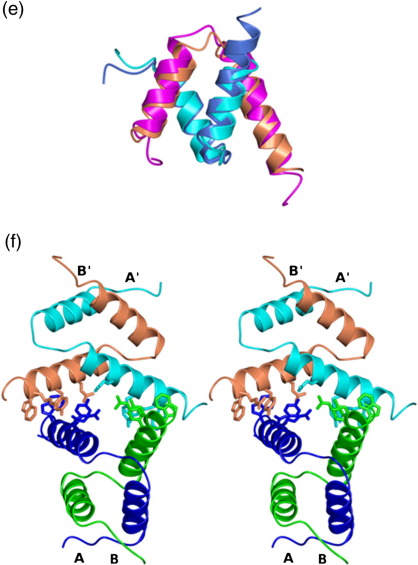
Crystal structures of the domains of SinR. (a) Stereo ribbon representation of the SinR(1–69) dimer coloured by chain and with the atoms of residues at the dimer interface shown in ball-and-stick format and non-carbon atoms coloured by atom type. Hydrogen-bonding interactions are shown as broken lines. The interface includes one and three histidine residues from the polyhistidine purification tags attached to the N-termini of chains A (green) and B (blue), respectively. (b) Overlay of the structure of the SinR(1–69) dimer in blue and the bacteriophage 434 Cro protein dimer in cyan from the complex of the latter with a  20-bp operator OR1 (light green). (c) Electron density shown in stereo and displayed on the structure of SinR(74–111) in the region of the dimer–dimer interface. The protein atoms are coloured by chain, and the 2*F*_o_ − *F*_c_ map is displayed at the 1.1 σ level. (d) Stereo ribbon representation of the SinR(74–111) dimer. The chains are coloured blue and green, respectively, and the chain termini are labelled. The side chains of residues contributing to the intermolecular hydrophobic core (black asterisks in Fig. 1b) are shown in cylinder format and coloured by chain. (e) Superposition of the SinR(74–111) dimer onto the heterodimerisation domain of SinR–SinI. The chains of the SinR(74–111) dimer are coloured light blue and blue; the SinR and SinI chains from the heterodimer are coloured coral and cyan, respectively. The structures have an rmsΔ of 0.99 Å over their corresponding backbone atoms. (f) The SinR(74–111) tetramer in stereo coloured by chain is formed from two dimers, one shown in cyan and coral and the other, in blue and green. Dimer–dimer interactions involve the C-termini of all four chains. Residues in the SinR(74–111) tetramer interface are shown as sticks. The tetramer is stabilised by salt bridges between the side chains of Glu97 and Arg105, and π–π stacking interactions between the side chains of Tyr101 and Trp104 from each chain.

Analysis of the molecular packing in the SinR(1–69) crystal lattice using the protein interaction server PISA^21^ revealed a significant interface between molecules A and B, formed around a non-crystallographic 2-fold symmetry axis (Fig. 4a). The intermolecular interactions involve the α3–α4 loops, which approach one another, forming main-chain and side-chain hydrogen bonds at residues 40–44, and the α5 helices at the C-terminus. The interactions of the two protomers lead to the burial of 1120 Å^2^ of what would otherwise be accessible surface area, and PISA predicts the dimer to be stable.^21^ The extent of surface area buried is towards the bottom end of that found in permanent dimer interfaces in proteins,^22^ and dimers were not observed at the lower protein concentrations used in the SEC-MALLS experiment. Moreover, 20% of this buried surface area is contributed by residues of the amino-terminal polyhistidine purification tags packing onto residues of helix α4 of the partner subunit (Fig. 4a).

This dimer is nevertheless very likely to have physiological significance because of its evident similarity to dimers of the Cro protein from bacteriophage 434.^23^ Comparison of the SinR(1–69) dimer with the Cro dimer in its complex with a  20-bp duplex O_R_1 operator DNA reveals that 116 equivalent C^α^ atoms can be superimposed with a positional rmsΔ of 2.85 Å (Fig. 4b). The subunit interfaces and their juxtaposition to the DNA-interacting HTH motifs in the two chains are well conserved. Overall, we conclude that the subunit interactions are too weak to stabilise dimers of SinR(1–69) at low protein concentrations but that they are sufficiently strong to support dimer formation at higher SinR(1–69) concentrations and to mediate interactions of the DNA-bonding domains in the intact SinR tetramer. In the latter instance, the effective concentration of the DNA binding domains is elevated because the chains have already been brought together by the interactions of the C-terminal domains (see below).

### SinR(74–111) is the tetramerisation domain

The C-terminal domain of SinR spanning residues 74–111 was analysed by SEC-MALLS. As shown in Fig. 2c, this domain is resolved as a single peak on the size-exclusion column, and the associated molecular mass was calculated to be 19 kDa, close to that expected for a tetramer, 19.9 kDa. We conclude from this clear result that dimerisation and tetramerisation determinants in SinR reside in the C-terminal domain.

To explore the structure of the SinR tetramer, we grew crystals of native SinR(74–111), and data were collected to a nominal resolution of 2.3 Å (Table 1). The diffraction pattern obtained from these crystals was of poor quality with streaky spots; nevertheless, the intensities could be indexed and integrated (Table 1). Molecular replacement using the native data set and a search model comprising residues 74–108 of SinR and 3–38 of SinI from the SinR–SinI coordinate set 1B0N gave a satisfactory solution, identifying the space group as *P*6_1_22. However, structure refinement stalled at *R* and *R*_free_ values of 28.4% and 43.5%, respectively. Although we were unable to refine the structure further, for reasons explained in Materials and Methods, we are confident that it is essentially correct. In particular, the quality of the electron density maps in the vicinity of the molecular interfaces, the subject of interest here, is good (Fig. 4c).

The structure allowed us to define the arrangement of the chains in the asymmetric unit. The two SinR(74–111) chains, A and B, each consisting of two α-helices (Fig. 4d), form the anticipated two-helical hooks that interlock in a four-helix bundle, reminiscent of that formed by SinI and the C-terminal domain of SinR in the SinR–SinI complex (Fig. 4e). Following least-squares superposition, the positional rmsΔ is 1.2 Å for 65 C^α^ from two chains. The intermolecular hydrophobic core between the A and the B protomers in which 2800 Å^2^ of accessible surface area is buried is composed of residues Trp78, Leu81, Val82, Ala85, Met86, Val90, Phe95, Phe98 and Leu99 from each protomer. A tetramer is generated through the interactions of two such dimers about a 2-fold crystallographic symmetry axis (Fig. 4f). The SinR(74–111) dimers interact through their C-termini, placing their N-termini distal to the tetramer-forming interface. Interactions between the two dimers are mediated by residues Glu97, Tyr101, Trp104 and Arg105 from all four chains interacting in two clusters and burying a total surface area of 1100 Å^2^, as shown in Fig. 4c and f. These residues, which project from the same face of the α7 helix, form intermolecular aromatic stacking interactions (Trp104 and Tyr101) and salt bridges (Glu97 and Arg105). Each is well conserved in orthologous proteins from *Bacillus* species.

## Discussion

Copies of the 7-bp SinR DNA binding consensus sequence are found in different numbers and arrangements at SinR-regulated promoters, suggesting the possibility of binding in a variety of orientations and valencies.^7^ Fluorescence anisotropy results presented here show that purified SinR binds to short oligonucleotide duplexes containing pairs of SinRBMs in inverted repeat and tandem repeat orientations as well as to a duplex containing just a single SinRBM. SinR binds with 10-fold higher affinity to the inverted repeat duplex than to the other two sequences. This suggests that SinR is adapted to binding DNA elements that contain 2-fold rotational symmetry. As binding to the tandemly repeating sequence is no tighter than that to the single-site substrate, it is likely that SinR is binding specifically to one of the SinRBMs and nonspecifically to the other.

The length of the oligonucleotides used in this study would allow only two of the DNA binding domains of the SinR tetramer to bind to each duplex. SinR's affinity for the inverted repeat DNA duplex is low (*K*_d_ value of 0.35 μM) for a repressor–operator interaction. The presence of more than two SinRBMs at the *eps* and *yqxM* promoters, which are strongly regulated by SinR, suggests the possibility of generating higher affinity through DNA binding to three or four of the subunits in the tetramer. The SEC-MALLS experiment shown in Fig. 2b suggests that higher-order binding does take place. Here, SinR tetramers and DNA duplexes were mixed in a 1:1 molar ratio. Assuming independent binding of each duplex to the tetramer, SinR_4_–DNA_2_ to SinR_4_–DNA complexes would be present in a 1:2 concentration ratio. Consistent with this expectation, the experimental molecular mass value for the SinR–DNA complex was 74 kDa, between the expected molecular mass values of the SinR_4_–DNA (67 kDa) complex and the SinR_4_–DNA_2_ (81 kDa) complex. At the *eps* and *yqxM* promoters, simultaneous binding to SinR_4_ of greater than two SinRBMs would, of course, require DNA looping (see below).

### The SinR tetramer

**Fig. 5:**
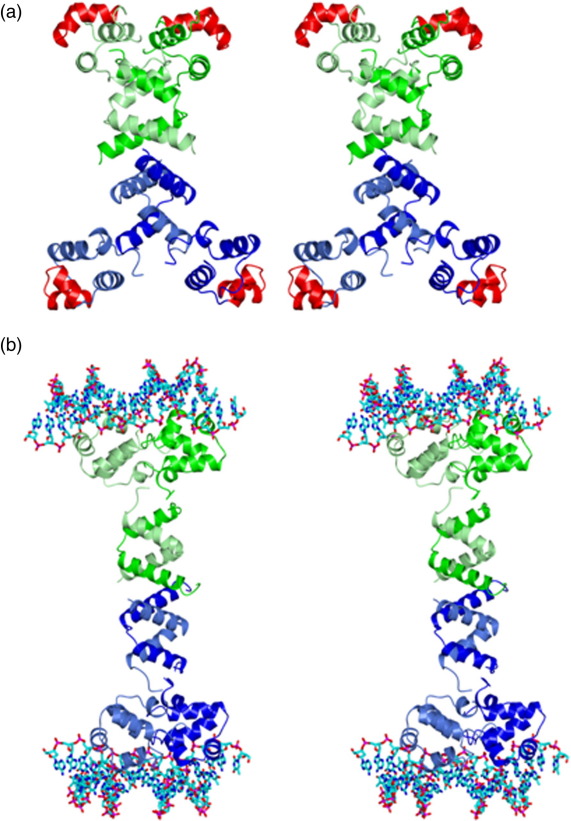

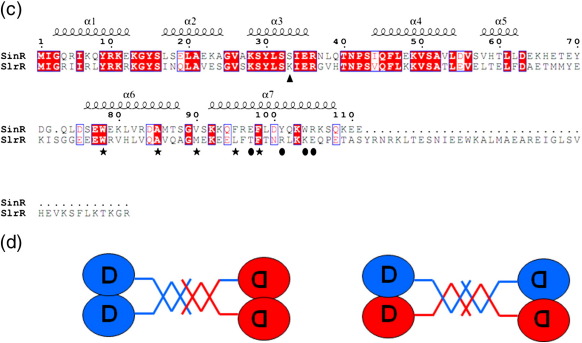
The SinR tetramer and SlrR comparison. (a) Stereo model of a SinR tetramer generated by superimposing the coordinates of SinR from the SinR–SinI complex onto each of the four chains of the SinR(74–111) tetramer. The chains of one dimer are coloured light green and green, while those of the other chain are in light blue and blue. The HTH motif is coloured red for all four chains. The exact position of the N-terminal domains in relation to the rest of the protein is expected to be variable due to the flexible linker connecting the domains. (b) Stereo model of a SinR tetramer bound to DNA. Pairs of DNA binding domains from the model generated above were brought together to form dimers matching the molecular packing in the SinR(1–69) crystal. The DNA duplexes are taken from the Cro–DNA (PDB entry 3CRO) complex following least-squares superposition of Cro protein C^α^ atoms onto the SinR(1–69) dimer. (c) A sequence alignment of SinR and SlrR. Identical residues are highlighted red, with similar residues boxed. Residues of the intermolecular hydrophobic core in SinR are indicated with asterisks, and those involved in dimer–dimer interactions are denoted by filled ovals. The alignment was created using ClustalW and ESPript.^17^ (d) SinR–SlrR complexes based on a heterodimer of dimers (left) or a dimer of heterodimers (right) models.

The hydrodynamic data and the crystal structures of the N- and C-terminal domains of SinR presented here give insight into the organisation of the full-length tetramer. The SinR(74–111) tetramer is a dimer of dimers in which the C-terminal helices mediate both dimer and tetramer formation. In generating a model of the full-length SinR tetramer, we first superposed residues 74–108 of the SinR chains from the SinI–SinR heterodimer coordinate set onto the corresponding residues of each of the two chain in the asymmetric unit of the SinR(74–111) crystal. These superpositions led to rmsΔ values in the positions of 35 equivalent C^α^ atoms of 1.2 Å and 1.4 Å for chains A and B, respectively. In the SinR dimers generated by this rigid-body superposition, there are relatively minor side-chain clashes between the DNA binding domains and helix α6 in the oligomerisation domain of the partner subunit. These can be relieved by manual modelling assuming flexibility in the domain juxtaposition within each SinR chain. Such flexibility is implied by the disorder in the interdomain linker (residues 70–73) in the SinI–SinR complex.^15^ The tetramer, generated by applying the crystallographic symmetry operator to the modelled SinR dimer, is shown in Fig. 5a.

The C-terminal helical bundle is positioned in the centre of the assembly, with the N-terminal DNA binding domains radiating from this core. The implied disorder in the linker segment connecting the two domains described above is expected to confer considerable flexibility in the juxtaposition of the four DNA binding domains. In the model, all four subunits are available to bind DNA, although occupancy of greater than two of the binding sites would require DNA looping. In each of the SinR dimers, the chains are related by an approximately 2-fold rotational axis of symmetry. These dimers would be expected to bind preferentially to substrates that share this symmetry, accounting for the higher affinity for the oligonucleotide duplexes with the inverted repeat arrangement of the SinRBMs. However, the flexibility referred to earlier could allow relative rotation of one DNA binding domain relative to the other, enabling the dimer to bind to tandemly arranged sequences. This capacity to bind tandem and inverted repeat sequences has also been proposed for Spo0A.^24^

In the model shown in Fig. 5a, the pairs of DNA binding HTH motifs (coloured red) in neighbouring subunits are too far apart to bind simultaneously to adjacent major grooves on one face of the DNA in the manner characteristic of bacteriophage λ-type repressor protein–DNA interactions.^25^ However, the intermolecular packing of the DNA binding domains in the SinR(1–69) crystal creates a dimer very similar to the bacteriophage 434 Cro protein dimer (Fig. 4b). Like SinR, Cro binds to DNA sequences approximately 20 bp in length and composed of an inverted repeat of a DNA half-site sequence. By manual modelling, we moved the DNA binding domains of the upper and lower pairs of subunits in the model of Fig. 5a together, so that they are juxtaposed as they are in the SinR(1–69) crystal. This required a relatively large DNA binding domain rotation of approximately 55° but only a small movement of the residues at the C-terminus of this domain, which form the link to the tetramerisation domain. Finally, we displayed the OR1 DNA sequence from the Cro–DNA complex [Protein Data Bank (PDB) entry 3CRO] on the model following least-squares superposition of the Cro and SinR(1–69) dimers (Fig. 5b). In this model, the two oligonucleotides are separated by the length of the long axis of the SinR tetramer, which spans ∼ 100 Å. The SinR binding motifs upstream of the *epsA-O* and *yqxM-sipW-tasA* operons span 80–100 bp (Fig. 2a), suggesting the possibility of DNA looping accompanying SinR binding. This would allow SinRBM occupancy of three or even all four of the DNA binding domains. The possibility of DNA looping at the *yqxM* promoter has been raised previously by Chu *et al*.^7^ Using a  231-bp *PyqxM* fragment, these authors saw multiple electrophoretically retarded species upon incubation with SinR, consistent with the presence and utilisation of multiple binding sites.

### Implications for SlrR interactions

SinR is a tetrameric protein that inhibits the expression of the biofilm genes of the *epsA-O* and *yqxM-sipW-tasA* operons. This repression is relieved in the presence of SinI, which disassembles the SinR tetramer with concomitant formation of SinI–SinR heterodimers. Recent studies have shown that SinR has a second partner, the SinR-like protein SlrR.^27^ SlrR plays an important role in biofilm formation, as it determines whether cells grow in a planktonic motile state or whether they grow as long chains in a sessile state. Chaining requires the repression of (i) genes encoding autolysins, which break down the cell wall following cytokinesis, allowing daughter cells to be separated, and (ii) flagellar genes associated with motility. In a fascinating addition to the SinR repertoire, SlrR binds to SinR so as to “repurpose” the latter as a repressor of autolysin and motility genes.^19,26^ Thus, the SlrR–SinR complex binds to and inhibits transcription from the *lytABC* and *lytF* promoters, which direct autolysin gene expression,^19^ a repressor function possessed by neither SinR nor SlrR alone.

As shown in Fig. 5c, SlrR exhibits sequence homology to SinR, which is particularly high in the N-terminal DNA binding domain. There is a notable difference at residue 33 where Ser in SinR is replaced by Lys in SlrR. Residue 33 occurs in the recognition helix component of the HTH, and this residue substitution may account for the altered consensus DNA binding sequence that has been put forward for SlrR. This residue change is interesting, as an Ala-for-Lys residue difference is observed at this position on the recognition helices of the CI and Cro repressors of bacteriophage λ.^25^ In the λ system, this residue substitution is important in changing the relative affinities of the two proteins for the tripartite operators O_L_ and O_R_. The strong parallels between SinR and phage repressors have been noted before.^15,27^

The sequence similarity extends beyond the DNA binding domain and to residues 70–110, which encompass the oligomerisation domain of SinR, which in turn is homologous to SinI (Fig. 5c). One can anticipate, therefore, that SlrR forms dimers and perhaps even tetramers. Although the stoichiometry of the SlrR–SinR complex is not known, one possibility is that the SinR tetramer dissociates into dimers, which then associate with SlrR dimers in the formation of heterotetramers (Fig. 5d, left). This would lead to the prediction that the tetramer might bind to pairs of inverted repeat DNA sequences, one constituted of two SinR consensus sequences and the other, of two SlrR consensus sequences. This may occur, but the residues involved in dimer–dimer interactions in SinR are not conserved in SlrR (Fig. 5c). Another possibility is that the SlrR–SinR complex is formed through more intimate heterodimer interactions analogous to the associations of SinR and SinI. This is possible, as the pattern of hydrophobic residues involved in SinR–SinI and SinR–SinR interactions is largely conserved in SlrR. These SinR–SlrR heterodimers, as well as heterotetramers if there is further association (Fig. 5d, right), would be expected to bind to a pair of consensus sequences in inverted orientation, consisting of one SinR binding half-site and one SlrR binding half-site. It is rather easier in this scenario to explain why the individual repressors SinR and SlrR do not bind to these operators.

These predictions can be tested by further experiment into this system of simple proteins that generate complexity through their interactions.

## Materials and Methods

### SinR expression and purification

The coding sequences of SinR (111 residues) and two fragments encompassing residues 1–69 and 74–111 were amplified by PCR using primers listed in Supplementary Table S1 and cloned into a modified pET28 vector using ligation-independent cloning.^28,29^ SinR and SinR(74–111) with a cleavable hexahistidine tag and SinR(1–69) with a non-cleavable hexahistidine tag were produced in *Escherichia coli* BL21(DE3) cells grown in autoinduction media at 30 °C for 24 h. Cells were harvested by centrifugation and lysed by sonication in a buffer containing 20 mM Na_2_HPO_4_, 0.5 M NaCl and 30 mM imidazole, pH 8.5. The cell debris was removed by centrifugation, and the supernatant was loaded on to a  1-ml HisTrap HP nickel affinity column. This column was developed with a  30-500 mM imidazole gradient, with protein eluting between 100 and 300 mM imidazole. Following buffer exchange into 25 mM Tris (pH 8.5), 200 mM NaCl, 1 mM DTT and 0.5 mM ethylenediaminetetraacetic acid (EDTA), the SinR and SinR(74–111) histidine tags were cleaved by overnight incubation with human rhinovirus 3C protease at a ratio of 1:100 (protease to protein). This is expected to generate SinR and SinR(74–111) bearing a residual N-terminal Gly-Pro-Ala sequence. The SinR(1–69) protein retains the N-terminal GSSHHHHHH tag. The proteins were purified further by size-exclusion chromatography using a HiLoad 16/60 Superdex 75  pg column. Protein was stored at − 80 °C.

### SinI expression and purification

The coding sequence of SinI (57 residues) was amplified by PCR using primers listed in Supplementary Table S1 and cloned into a modified pET28 vector, as described above for SinR. SinI was produced in *E. coli* BL21(DE3) cells grown in autoinduction media at 30 °C for 24 h. Cells were harvested by centrifugation and lysed by sonication in a buffer containing 20 mM Na_2_HPO_4_, 0.5 M NaCl and 30 mM imidazole, pH 8.0. Nickel affinity chromatography, histidine tag cleavage and size-exclusion chromatography were performed as described for SinR, using buffers at pH 8.0. Purified protein was stored at − 80 °C.

### Annealing DNA oligonucleotides

Oligonucleotides, purified by HPLC, were purchased from Eurofins MWG Operon. Complementary oligonucleotides were incubated in a 1:1.1 molar ratio in a buffer composed of 25 mM Tris (pH 8.5), 200 mM NaCl, 1 mM DTT and 0.5 mM EDTA. The oligonucleotides were heated to 90 °C for 5 min to ensure full denaturation, followed by slow cooling to room temperature. For SPR experiments, one DNA strand was 5′ labelled with biotin. For fluorescence anisotropy experiments, one DNA strand was 5′ labelled with HEX. These labelled strands were annealed to unlabelled complementary oligonucleotides, as described above.

### Size-exclusion chromatography with multi-angle laser light scattering

A Wyatt Dawn HELEOS-II 18-angle light-scattering detector and Wyatt Optilab rEX refractive index monitor linked to a Shimadzu HPLC system and SPD20A UV/Vis detector were used for SEC-MALLS. A Superdex 75 HR 10/30 size-exclusion column was attached to the HPLC and equilibrated in a running buffer consisting of 25 mM Tris (pH 8.5), 200 mM NaCl and 0.5 mM EDTA. An SIL-20A Autosampler was used to inject  100-μl samples of 1 mg ml^− 1^ SinR, 19 μM DNA or a mixture of the two. Data were analysed with the Astra software using *dn*/*dc* values of 0.186 for protein and protein–DNA complexes and 0.168 for DNA.^30,31^ The experiment was repeated as described above using 1 mg ml^− 1^ SinR(74–111), 1 mg ml^− 1^ SinR(1–69), 1 mg ml^− 1^ SinR, 1 mg ml^− 1^ SinI and an equimolar mixture of SinR and SinI.

### Fluorescence anisotropy

Fluorescence anisotropy experiments were carried out using a Horiba FluoroMax-3 spectrophotometer.^20^ HEX-DNA (5 nM) was prepared in 1 ml of 10 mM Hepes (pH 8.0), 100 mM NaCl, 1 mM DTT and 1 mM EDTA in a  1-ml Hellma Quartz cuvette. The excitation and emission wavelengths used were 530 nm and 580 nm, respectively, with a  10-nm slit width. SinR was titrated into the cuvette, and the anisotropy was measured after each addition. Ten measurements, with an integration time of 1 s, were taken for each sample following each addition, and these were averaged. All experiments were carried out at 25 °C. The data were analysed using the Scientist 3.0 software from Micromath to determine binding constants. The average value of *K*_d_ from three runs was calculated with errors estimated by standard deviation.

### Surface plasmon resonance

SPR was carried out using a Biacore T100, with an SA sensor chip. Biotinylated DNA, purchased from Eurofins MWG Operon, was annealed to the complementary unlabelled DNA as previously described and immobilised on the sensor chip to a level of 50 response units. The running buffer was 10 mM Hepes (pH 8.0), 150 mM NaCl, 3 mM EDTA and 0.05% P20, and the regeneration buffer was 1 M NaCl and 50 mM NaOH. SinR was injected over the chip at a range of concentrations, with a duplicate of the highest concentration. The experiment was run with the following parameters: five start-up cycles; flow rate, 30 μl min^− 1^; injection, 240 s; dissociation, 600 s; and regeneration, 30 s. A reference cell was used with no DNA immobilised. Data were analysed using the Biacore T100 evaluation software.

### SinR(1–69) crystallisation, data collection and structure solution

Crystals of SinR(1–69) were grown using the vapour diffusion method in hanging drops formed by mixing 1 μl 7 mg ml^− 1^ SinR(1–69) with 1 μl of 0.1 M Tris (pH 8.5), 27% w/v polyethylene glycol (PEG) 3350 and 5% glycerol. Rod-shaped crystals appeared after 2 days. Crystals were soaked in mother liquor containing 22% glycerol and rapidly vitrified in liquid nitrogen. X-ray diffraction data were collected to 1.9 Å resolution at ESRF and processed and reduced using the HKL2000 and SCALEPACK packages.^32^ The crystals belong to space group *P*2_1_2_1_2_1_ with unit cell parameters *a* = 34.97 Å, *b* = 45.33 Å, *c* = 85.25 Å and α = β = γ = 90°. Assuming two molecules in the asymmetric unit the estimated solvent content is 36%.

The structure was solved by molecular replacement in the program MOLREP^33^ using the DNA binding domain (residues 1–69) coordinates from the crystal structure of the SinR–SinI complex (PDB code 1B0N) as the search model. Two solutions, related by 2-fold rotational symmetry, were obtained. The structure was refined using maximum-likelihood methods implemented in REFMAC^34^ interspersed with sessions of manual modelling using Coot^35^ to a final *R*-factor of 0.20 and an *R*_free_ of 0.25.

### SinR(74–111) crystallisation, data collection and structure solution

SinR(74–111) crystals grew somewhat irreproducibly from solutions containing 18% PEG monomethyl ether 2000, 0.16 M Tris (pH 8.5), 0.01 M Ni(II) chloride and 0.1 M MgCl_2_ at a protein concentration of 7 mg ml^− 1^, using the hanging-drop vapour diffusion method. After 1 week, drops containing crystals were transferred to a solution of mother liquor containing 27% PEG monomethyl ether 2000. Crystals were left for one more week before rapid cooling in liquid nitrogen. The crystals diffracted to 2.3 Å on beamline I03 at the Diamond synchrotron. The diffraction data indicated that the crystals belong to space group *P*6_1_22 or its enantiomorph *P*6_5_22 with unit cell parameters *a* = 36.12 Å, *b* = 36.12 Å, *c* = 250.34 Å, α = 90°, β = 90° and γ = 120°. Assuming that there are two chains in the asymmetric unit, the packing density is 2.6 Å^3^ Da^− 1^, and the solvent content is 53%. Selenomethionine (SeMet) SinR(74–111) crystallised under the same conditions and in the same space group as the native protein, with unit cell lengths of *a* = 36.89 Å, *b* = 36.89 Å and *c* = 248.56 Å. Although the cell dimensions and point group of the two crystals were very similar, the native and derivative crystals were not isomorphous, with data scaling giving a mean isomorphous difference (*R*_iso_) value of ∼ 55%.

The native structure was initially solved by molecular replacement using the heterodimerisation domain (residues 74–108 of SinR and 3–38 of SinI) of the SinR–SinI complex (PDB code 1B0N) as a search model. The solution clearly identified the space group as *P*6_1_22 rather than *P*6_5_22, and automated refinement reduced the *R*-factor to 0.41 (*R*_free_ = 0.51). The SinI component was replaced by SinR using the alignment shown in Fig. 1b, and further refinement and rebuilding lowered the *R*-factor to 0.28 (*R*_free_ = 0.43). These high values presumably reflect the poor-quality data. Nevertheless, the maps were clear enough to build missing side chains and the important tetramerisation interface comprising residues 97–105 of the two chains (Fig. 4c). The conformation of this region of the structure was confirmed by excluding residues of interest from the model and inspecting difference maps following cycles of refinement. In contrast, the quality of the electron density was poor for the Gly-Pro-Ala tag and residues 74–77 at the amino terminus and residues 108–111 at the C-terminus of both chains.

Analysis of the anomalous measurements for the SeMet crystals identified the positions of the two expected selenium atoms, plus a third anomalous scatterer (which we later modelled as a nickel ion introduced from the crystallisation solution), but the multiwavelength anomalous dispersion-phased electron density maps were of disappointing quality and not interpretable.

The refined model generated with the native data was used for a molecular replacement search with the SeMet peak data set. This gave a solution very similar to that obtained with the native data, but with a relative rotation of 2.5°, explaining the lack of isomorphism between the native and the derivative data sets. The associated rotation axis is close to that generating the SinR tetramer, so that the tetramer interface is conserved in the two crystal forms. Encouragingly, anomalous difference maps calculated with the SeMet data and these molecular replacement phases clearly revealed the three anomalous scatterers (two Se and Ni). This suggests that the native structure is essentially correct.

For interest, a postmortem phase analysis of the experimental phases against the calculated phases for the SeMet crystal was carried out and gave an overall phase error of ∼ 70°. None of the experimental phase improvement techniques were successful, generating maps with poor continuity. This may be due to the very incomplete low-resolution data, a consequence of the crystal morphology and the elongated unit cell.

### Accession numbers

The coordinates and structure factors for SinR(1–69) and SinR(74–111) have been deposited with the PDB with accession codes 3QQ6 and 2YAL, respectively.

The following is the supplementary material related to this article.

Supplementary Table S1 Oligonucleotide primers used in this work for amplification of coding sequences by PCR

## Figures and Tables

**Table 1:** Data collection and refinement statistics

	Native SinR(1–69)	Native SinR(74–111)	SeMet		
			Peak SinR(74–111)	Inflection SinR(74–111)	Remote SinR(74–111)
*Data collection*					
X-ray source	ESRF, ID23-1	DLS, I03		DLS, I03	
Wavelength (Å)	1.0039	0.9789	0.9801	0.9804	0.9763
Collection temperature (K)	120	120		120	
Resolution range (Å)	50.00–1.90	50.00–2.28	50.00–2.70	50.00–2.90	50.00–3.00
Space group	*P*2_1_2_1_2_1_	*P*6_1_22		*P*6_1_22	
Unit cell parameters					
*a*, *b*, *c* (Å)	34.97, 45.33, 85.25	36.10, 36.10, 250.32		36.98, 36.98, 248.60	
α, β, γ (°)	90, 90, 90	90, 90, 120		90, 90, 120	
Number of unique reflections, overall/outer shell^a^	10,989/539	4405/200	3176/124	2642/107	2570/112
Completeness (%), overall/outer shell^a^	98.2/98.5	85.7/42.4	95.6/83.8	96.5/88.4	97.1/.98.2
Redundancy, overall/outer shell^a^	4.5/4.6	12.5/7.6	8.8/6.3	7.3/6.8	7.5/6.6
〈*I*〉 〈/σ(*I*)〉, overall/outer shell^a^	19.6/4.9	59.6/2.4	53.2/4.0	54.8/6.3	42.5/3.8
*R*_merge_^b^ (%), overall/outer shell^a^	5.6/29.1	6.7/38.5	8.2/31.1	6.1/23.8	8.9/36.3

*Refinement and model statistics*					
Resolution range (Å)	42.62–1.90	41.72–2.20	32.0–2.66		
*R*-factor^c^ (*R*_free_^d^)	0.204 (0.255)	0.283 (0.435)	0.312 (0.489)		
Reflections (working/free)	10,411/524	4188/173	3218/79		
Outer-shell *R*-factor^c^^,^^e^ (*R*_free_^d^)	0.245/0.249	0.44/0.54	0.29/0.51		
Outer-shell reflections^e^ (working/free)	727/23	74/6	151/7		
Molecules/asymmetric unit	Chain A His_2_ 1–67	Chain A GPA 74–108	Chain A GPA 74–108		
	Chain B His_4_ 1–64	Chain B GPA 74–108	Chain B GPA 74–108		
		Chain M Ni^2+^	Chain M Ni^2+^		
Number of protein non-hydrogen atoms	1112	654	654		
Number of water molecules	78	0	0		
rmsd from target^f^					
Bond lengths (Å)	0.021	0.20	0.013		
Bond angles (°)	1.793	1.80	1.76		
Average *B*-factor (Å^2^)	28.4	82.7	94.9		
Ramachandran plot (%)^g^	98.5/1.5/0	93.1/4.1/2.7	87.7/2.7/9.6		

DLS, Diamond Light Source.

a The outer shell corresponds to 1.93–1.90 Å for Native(1–69), 2.36–2.28 Å for Native(74–111), 2.75–2.70 Å for Peak, 2.95–2.90 Å for Inflection and 3.05–3.00 Å for Remote.

b *R*_merge _= ∑_*hkl*_∑_*i*_|*I*_*i*_ − 〈*I*〉|*/*∑_*hkl*_∑_*i*_〈*I*〉 where *I*_*i*_ is the intensity of the *i*th measurement of a reflection with indexes *hkl* and 〈*I*〉 is the statistically weighted average reflection intensity.

c *R*-factor = ∑||*F*_o_| − |*F*_c_||*/*∑|*F*_o_| where *F*_o_ and *F*_c_ are the observed and calculated structure factor amplitudes, respectively.

d *R*_free_ is the *R*-factor calculated with 5% of the reflections chosen at random and omitted from refinement.

e Outer shell for refinement corresponds to 1.951–1.901 Å for Native(1–69), 2.36–2.30 Å for Native(74–111) and 2.76–2.70 Å for Peak.

f rmsΔ of bond lengths and bond angles from ideal geometry.

g Percentage of residues in preferred/allowed/disallowed regions of the Ramachandran plot, according to PROCHECK.
