# Supplementary material for: Structure and Organisation of SinR, the Master Regulator of Biofilm Formation in Bacillus subtilis
Source: J Mol Biol. 2011 Aug 19;411(3-26):597–613. doi: 10.1016/j.jmb.2011.06.004 (PMC3163267; doi:10.1016/j.jmb.2011.06.004)
Supplement: Supplementary Table S1 — Oligonucleotide primers used in this work for amplification of coding sequences by PCR [file mmc1.doc]

## Supplementary Table S1. Oligonucleotide primers used in this work for amplification of coding sequences by PCR

| **Primer^a^** | **Primer Sequence 5’→ 3’** |
| --- | --- |
| SinRF- SinR forward | CACCACCACCACATGATTGGCCAGCGTATTAAACAATACCGTAAAG |
| SinRR- SinR reverse | GAGGAGAAGGCGCGTTACTCCTCTTTTTGGGATTTTCTCCATTTTTG |
| SinRFC-SinR (3C) forward | CCAGGGACCAGCAATGATTGGCCAGCGTATTAAACAATACCGTAAAG |
| NSinRR-SinR (1-69) reverse | GAGGAGAAGGCGCGTTATTCGGTTTCATGTTTCTCATCGAGCAAAGTATG |
| CSinRF-SinR (74-111) (3C) forward | CCAGGGACCAGCAATCgatagtgaatgggagaaattggttcgcgatgc |
| SinIFC-SinI (3C) forward | ccagggaccagcaatgaagaatgcaaaacaagagcactttgaattgg |
| SinIR- SinI reverse | gaggagaaggcgcgttagaaaggatttacggtatgacttctggctgc |

^a^ Forward primers marked with 3C allow incorporation of the DNA product into pET-YSBLIC-3C, for fusion to a sequence encoding a 3C cleavage site. All other forward primers are designed for cloning into pET-YSBLIC. Underlined regions represent the LIC specific sequence.
